# Taking a Strengths‐Based Approach to Mental Health in Rural Communities: What Is the Evidence for Harnessing Strengths?

**DOI:** 10.1111/ajr.70061

**Published:** 2025-06-10

**Authors:** Annika Luebbe, Sandra Diminic, Zoe Rutherford, Hannah Roovers, Mikesh Patel, Harvey Whiteford

**Affiliations:** ^1^ The School of Public Health The University of Queensland Herston Queensland Australia; ^2^ Queensland Centre for Mental Health Research Wacol Queensland Australia

**Keywords:** assets, Australia, Canada, mental health, remote, resources, rural, socioecological framework, strengths, United States

## Abstract

**Objective:**

This study aimed to determine if rural community strengths identified in the literature have been causally linked to improved mental health and whether these strengths have been harnessed in interventions.

**Methods:**

A secondary analysis of a systematic review of literature from Australia, Canada and the USA identified 28 studies that proposed a conceptual relationship to improved mental health. Studies were categorised, their distribution across a socioecological framework was assessed, and evidence of causality was evaluated.

**Results:**

Among 28 studies, 24 were analytical and focused mainly on community strengths, with four interventional studies that addressed both personnel and community strengths. None established a causal relationship, including those that harnessed strengths in interventions.

**Conclusions:**

Although rural strengths have been associated with improved mental health, evidence on causality, effectiveness and mechanisms for harnessing remains limited. Strengthening the evidence base is critical to justify incorporating rural strengths into mental health commissioning.

**Summary**

- What is already known on the subject?
  ○ A deficit‐based lens is applied in rural mental health research.
  ○ However, assets and resources exist in rural communities.
  ○ Existing local strengths have the potential to be harnessed to improve mental health.
- What this paper adds?
  ○ Studies on rural mental health strengths tend to have a low level of evidence.
  ○ Strong causal relationships could not be established between strengths and improved mental health due to study designs.
  ○ Higher quality, interventional studies need to research rural strengths to enhance the evidence base.

## 1 Introduction

Rural mental health historically has been described in terms of deficiencies, often reflecting a narrative of poorer mental health outcomes and insufficient service provision [1]. Much of the rural mental health literature applies a deficit‐based approach to investigating the healthcare gap, ultimately perpetuating harmful narratives about rural life and healthcare [1, 2]. ‘Deficit‐based’ for the purposes of this study are approaches to research, service provision, or the broader discourse that surrounds rural health by focusing on: what is missing (e.g., workforce shortages [3]) or what is not ‘as good’ as or ‘worse than’ metropolitan areas (e.g., suicide rates increasing with remoteness [4, 5]), stigma associated with rural life (e.g., beliefs that rural people are less educated, or rural locations are unrewarding places to work [6]), and an emphasis on clinical solutions (i.e., funding models favouring clinical services and overlooking other nonclinical determinants of mental health [7]). Some of these ideas are undeniably concerning and may be a sign of, or a contributing factor to, rural mental health inequities [8]. Accordingly, research tends to focus on what is lacking in rural areas and solutions thus prioritise funding external clinicians, infrastructure and interventions designed and implemented through a metrocentric lens. These initiatives to date have had limited impact and do not ensure consistency or sustainability through complex funding cycles and with healthcare providers who stay for short or sporadic periods in rural areas [9]. This is not to say these solutions are not important or that clinicians are not required. Rather that the continued emphasis on deficiencies does not lend itself to acknowledging inherent protective factors, assets, resources and rural ways of functioning that may be helpful for preventing or managing mental health problems.

To better support the populations served, it is suggested that we understand what environmental and social factors contribute to better mental health. In a recent review of the social determinants of mental health and disorder, Kirkbride et al. [10] discuss negative influences on mental illness, such as disadvantage, adversity and isolation. They also recommend strategies that aim to optimise recovery, including the use of family interventions and social prescribing, that is, connecting those with mental disorders to sources of social support within local communities. Recent literature has reported that social prescribing strategies are well‐received and useful in rural areas but face barriers to uptake [11]. The Kirkbride review echoes these sentiments by suggesting that ‘despite its [gaining popularity outside of Australia], the evidence base lags behind practice, with studies currently lacking methodological rigour’ [10, 12]. Thus, requiring a greater evidence base. When discussing universal prevention, the examples identified include parenting programs, school‐based interventions to improve mental health literacy, and initiatives to reduce loneliness [10]. Though targeted and well‐meaning, these examples are still influenced by a deficit view, ‘what contributes to poor mental health and how can we reduce it?’, as opposed to ‘what resources, assets and strengths in a community promote good mental health, reduce the risk of mental ill‐health and how can we expand these?’.

Despite obvious health inequities, rural populations arguably have many existing assets and resources (strengths) that can be beneficial to managing and preventing mental illness [2]. Such strengths may be the result of connected and supportive communities, or creativity born out of necessity due to limited health facilities and workforce. Recent literature supports the incorporation of strengths‐based approaches in research [2] and proposes mapping existing community capital and assets [13]. Doing so could inform policy creation and improve mental health care provision in a way that is relevant and sustainable whilst acknowledging rural agency [13, 14, 15]. However, there is limited research that investigates how to effectively engage these strengths to improve the mental health of rural populations.

To understand what strengths, linked to mental health, have been identified in rural communities, the authors conducted a systematic literature review of Australian, Canadian and United States research [16]. This secondary study builds upon that work by conducting further analyses of the identified literature. For the review, the term ‘strengths’ referred to a resource or asset, inherent within a rural community that has the potential to promote or manage mental health (sometimes referred to in the literature as a protective factor) [2, 17]. In this use of the term, a strength is not an intervention or service introduced from outside the community, but encompasses the extent to which resources and processes within a community maintain and enhance both individual and collective well‐being [1, 18, 19]. The review extracted 61 papers that identified a variety of strengths that proposed a relationship with mental health [16]. Strengths were identified across the Rural Strengths Socioecological Framework continuum—spanning nature, the individual, interpersonal relationships, community, to lay and clinical personnel. Of the 61 papers, 28 proposed a conceptual link between a strength and improved mental health. However, the extent to which the conceptual link could be considered causal required further investigation.

This study is a secondary analysis of the aforementioned systematic literature review that identified rural mental health strengths [16]. This study aimed to (1) identify the causal links and examples of harnessing across the US, CAN and AUS in terms of a socioecological framework, (2) categorise the articles by study design and strength of evidence to determine if the strengths were causally linked to improved mental health and (3) explore examples of how strengths have been harnessed to improve mental health.

## 2 Methods

### 2.1 Primary Systematic Literature Review

**FIGURE 1:**
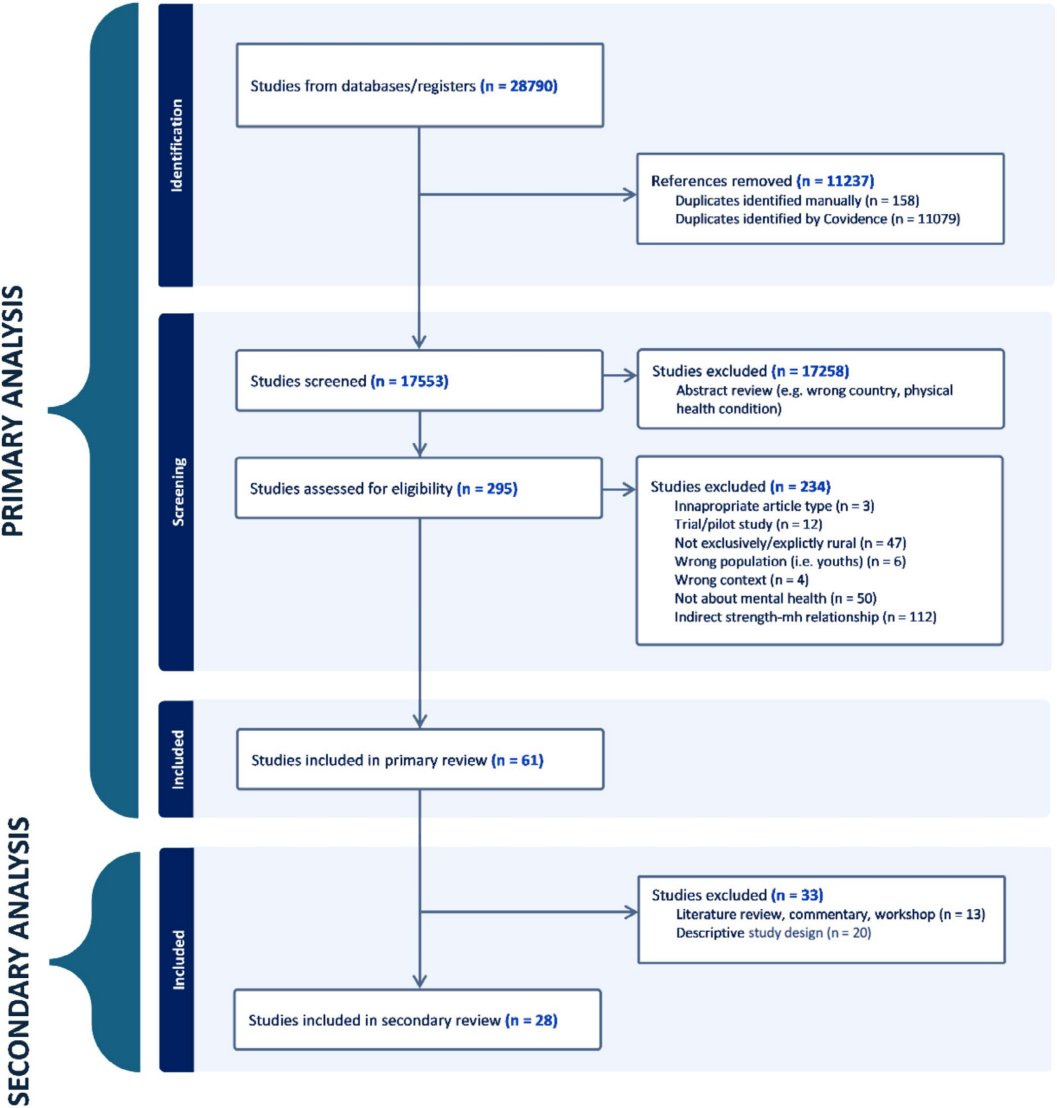
Primary and secondary analysis PRISMA flowchart.

A systematic review of literature was conducted on electronic databases PubMed, Scopus, CINAHL and PsycInfo using PRISMA (Preferred Reporting Items for Systematic Reviews and Meta‐Analyses) guidelines [20] (see Figure 1, primary analysis). The search strategy used a combination of broad MeSH/Subject terms using Boolean operators and text phrases relevant to rurality, mental health and strengths (see Appendix 1). The review included peer‐reviewed studies, reports and theses about mental health and strengths, published between January 1990 to October 2022 from rural populations in Australia, Canada and the United States of America. The scope of this review was contained to these three countries due to their similarities in geographic size, rural context, population dispersion, Indigenous populations and being primarily English speaking. Limiting to AUS, CAN and USA was necessary to manage the scope of the review. Systematic review software, covidence, was used to assist in the review process. The majority of screening was conducted by one reviewer (AL), and two additional reviewers (HP and MP) assisted in the final iteration of full‐text screening. All reviewers independently screened the full‐text reviews and were blinded to others' inclusions. Screening conflicts between reviewers were resolved through discussion to reach consensus before the final list of included articles was confirmed. Literature met the inclusion criteria when studies were exclusively rural, regional or remote, highlighted a conceptual link between mental health and strengths, investigated an adult and/or general population (not exclusively youth‐focused) and published in English. Ethics approval was not required for this systematic review.

The primary analysis extracted 61 applicable studies. Where possible, information about the literature was extracted, including rurality classification, identified strengths, strengths‐mental health relationship/conceptual link, study design, demographics, country, year and author. Studies were then thematically analysed using a mixture of reflexive, deductive and inductive methods. During this process, the Rural Strengths Socioecological Framework was developed—adapted from the US Department of Health socioecological framework [21] and informed by the results from the thematic analysis. Where strengths were identified in studies, they were categorised into the Framework's rural strength levels accordingly [16].

### 2.2 Secondary Analysis

The 61 studies identified from the primary analysis were examined further in this study. First, the study designs of each paper were assessed. This allowed for the research design and strength of evidence to be evaluated, and assisted in determining whether studies had conceptual links or could propose causal relationships between a strength and improved mental health [22].

Studies that were classified as descriptive [23] were excluded as this design did not provide the information necessary for relationships to be identified (see Figure 1, secondary analysis). The remaining studies were classified into one of two study designs: analytical studies [24] (where the conceptual relationship between a strength and improved mental health is identified, and a causal relationship may be proposed from the data), and interventional studies [25] (where participants are exposed to a strength, the outcome of this on mental health is measured, and a potential causal relationship may be established from the data).

Of the 61 initial studies, 28 remained that were analytical and interventional. The identified strengths of each paper were mapped to the five domains of the Rural Strengths Socioecological Framework [16] to assist in contextualising where strengths occur within the community. Each study was reviewed to determine its level of evidence on the National Health and Research Council (NHMRC) evidence hierarchy [26]. The hierarchy consists of five designations of levels of evidence according to the qualities of the research and how evidence was obtained, where ‘level IV’ is least strong and ‘level I’ is the strongest level of evidence (IV case series, III‐3 comparative study without concurrent controls, III‐2 comparative study with concurrent controls, III‐1 pseudo randomised control trial, II randomised control trial, systematic review of level II studies) [26].

## 3 Results

The 28 studies included in the secondary analysis originated from Canada (*n* = 5), the United States of America (*n* = 8) and Australia (*n* = 15), and used qualitative, quantitative and mixed‐method approaches [16].

### 3.1 Rural Strengths Socioecological Framework

**FIGURE 2:**
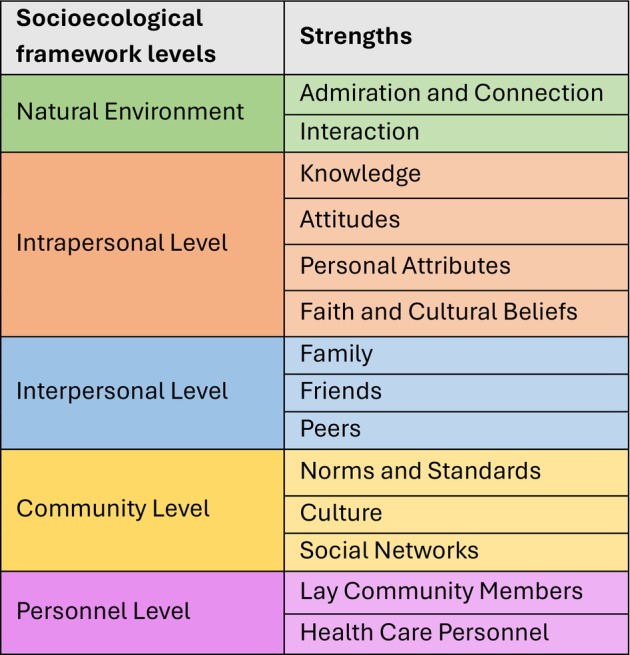
Rural Strengths Socioecological Framework (simplified).

Thematic analysis of the 28 studies identified where strengths existed across the five domains of the Framework (Figure 2). The Rural Strengths Framework aims to capture a holistic understanding of the many contributions to mental health throughout the extent of the rural environment [16].

### 3.2 Analytical and Interventional Study Designs

Of the 28 studies, 24 were classified as analytical and 4 as interventional in study design. The 24 papers classified as analytical studies varied in methodology, including focus groups, semi‐structured interviews, cross‐sectional and longitudinal surveys, photovoice analyses and evaluations of mental health programmes. These were primarily qualitative in nature, with few mixed‐method designs. Studies identified strengths from all domains of the Socioecological Framework, with a particular focus on community. Table 1 includes numerical identification of all studies and their relevant strengths and findings. Examples include: football clubs [2], men's sheds [4] and women's curling teams [11, 27] as community resources; connection to place and nature [1, 10, 13] and lakeshore settings [16] as beneficial for mental health; as well as social capital [6, 9] and community connectedness [10, 11, 12, 13, 23] as potential assets that could be harnessed to improve mental health (see Table 1).

**TABLE 1:** Study properties—study designs, strengths and overall findings.

No.	Title	Authors	Country	Year	Study design	Strength of evidence^a^	Strength studied	Findings
1	A community‐led design for an Indigenous Model of Mental Health Care for Indigenous people with depressive disorders	Farah Nasir et al. [27]	AUS	2021	Analytical	IV	Nature: connection to country Intrapersonal: spirituality, culture Community: connection to culture	‘Connection to culture and Country were described as inalienable Indigenous rights and as pathways to improved health and wellbeing outcomes’
2	Barriers and enablers to implementing mental well‐being programs through Australian rural football clubs‐A qualitative descriptive study	Hutchesson et al. [28]	AUS	2021	Analytical	IV	Community: football club Personnel: club members	‘One club had two mental health advocate positions, two had members trained in Mental Health First Aid and two had offered counselling to their members following a death by suicide of a club member through a chaplain, with one reporting that their chaplain has regular contact with the club. Dedicated mental health supports were seen as an important means (enablers) for encouraging people to reach out if they needed assistance’
3	Mental health first aid training for Extension agents in rural communities	Robertson et al. [29]	USA	2021	Interventional	IV	Intrapersonal: knowledge, mental health first aid training Community: cultural competence/understanding Personnel: rural extension agents	‘Over 60% of the agents reported using the skills learned from the MHFA training, and nearly 15% of agents reported having an encounter with someone in crisis since completing the MHFA training’ ‘Extension Agents stand in key positions to both help identify those at risk of suicide and provide them with support and information around mental health’
4	Men's Sheds and the experience of depression in older Australian men	Culph et al. [30]	AUS	2015	Analytical	IV	Intrapersonal: self‐worth Interpersonal: social relationships Community: men's sheds, social connectedness	‘The findings from this study support the notion that participation at Men's Sheds decreases self‐reported symptoms of depression’
5	Relationships between social connectedness and spirituality and depression and perceived health status of rural residents	Galloway and Henry [31]	USA	2014	Analytical	IV	Community: social connection Personnel: nurses	‘Social connectedness was a significant predictor of self‐reported depression…As social connectedness increased, depressive symptoms decreased’ ‘Nurses can also take the leadership role of forming coalitions to work on developing these practices. Incorporating already established rural institutions such as churches, schools and athletic events and obtaining buy‐in from rural community leaders can help assure the interventions are successful and meets the needs of the residents’
6	Financial strain, social capital and perceived health during economic recession: a longitudinal survey in rural Canada	Frank et al. [32]	CAN	2014	Analytical	III‐3	Community: social capital	‘Social capital related to less stress, better physical health, and fewer symptoms of anxiety and depression’
7	Mental health recovery and quilting: Evaluation of a grassroots project in a small, rural, Australian Christian church	Wilson [33]	AUS	2014	Interventional	IV	Community: social connectedness Personnel: mental health nurse	‘This project…demonstrates how a church faith community and mental health care can be combined and yield positive outcomes…[and] indicates that mental health professionals already established in their dual roles as church member and mental health professional can contribute in a unique way to the mental health of individuals within their usual church setting’ ‘This promoted acceptance in both directions and enhanced mental health recovery potential and shared community values’
8	Exploring mental health issues of rural senior women residing in southwestern Ontario, Canada: A secondary analysis photovoice study	Panazzola and Leipert [34]	CAN	2013	Analytical	IV	Intrapersonal: personal characteristics, resilience Interpersonal: family and friend support Community: community groups and resources, church setting Personnel: public health nurses	‘Two factors were found to positively affect these women's mental health: social and community resources, and personal characteristics and resources’ ‘Resiliency emerged as an important personal characteristic and was identified as having a positive effect on their mental wellbeing’
9	Determinants of mental health and well‐being within rural and remote communities	Kelly et al. [35]	AUS	2011	Analytical	IV	Intrapersonal: neuroticism, sense of belonging Community: community connectedness, social capital Interpersonal: personal social support	‘The chief determinants of current wellbeing were those reflecting individual level attributes and perceptions, rather than district‐level rural characteristics. This has implications for strategies to promote wellbeing within rural communities through enhancing community connectedness and combating social isolation in the face of major adversities such as drought’ ‘…indicating that higher wellbeing (and lower K‐10 scores) were associated with lower neuroticism, fewer adverse events, and higher social support’
10	Living in a rural community Is good for your health… or is it? Young women talk about rural living and their emotional and mental health	Jackson et al. [36]	CAN	2011	Analytical	III‐3	Nature: engagement, interaction, place attachment, connection to place Intrapersonal: safety, security and trust Community: social connectedness	‘Our research indicates that specific characteristics of the physical and social environments promote feelings and experiences of “connectedness”, thus contributing positively to emotional and mental health’ ‘The women in both communities spoke of feeling ‘connected’ to both the physical and social environments. they presented these experiences of connectedness as having positive implications for their emotional and mental health, because they were linked to feelings of joy, freedom, safety and security’
11	‘I can't imagine my life without it!’ Curling and health promotion: a photovoice study	Leipert et al. [37]	CAN	2011	Analytical	IV	Community: curling team, social connectedness Personnel: nurses	‘The findings reveal that curling facilitates social connections, enhances physical and mental health, and provides a valued and visible way to support rural life. Clearly, curling promotes the health and community life of rural women in significant ways’
12	Social capital and mental health among Indigenous Australians, new Australians and other Australians living in a coastal region	Berry [38]	AUS	2009	Analytical	IV	Intrapersonal: optimism, trust, sense of belonging Community: social connectedness	‘For all groups, higher levels of participation, more positive perceptions about participation, and greater cohesion were associated with less distress and more happy feelings’ ‘Greater social capital overall was strongly associated with less distress and more happy feelings, the former particularly for Aboriginal Australians’
13	Social networks and mental health among a farming population	Stain et al. [39]	AUS	2008	Analytical	IV	Nature: connection to place Interpersonal: community support Community: social connectedness	‘This study has highlighted the association between unique rural community characteristics and rural stressors (such as drought) and measures of mental health, suggesting the important mediating role of social factors and community characteristics’ ‘Community support…showed significant negative associations with K10 scores’
14	Religious beliefs, faith community involvement and depression: a study of rural, low‐income mothers	Garrison et al. [40]	USA	2004	Analytical	IV	Intrapersonal: religious beliefs Community: faith community, church	‘As hypothesised, both religious beliefs and faith community involvement were negatively related to depressive symptoms’
15	Religiousness/spirituality and subjective well‐being among rural elderly Whites, African Americans and Native Americans	Yoon and Lee [41]	USA	2004	Analytical	IV	Intrapersonal: spirituality, spiritual coping skills	‘This study found…a significant association between dimensions of religiousness/spirituality and subjective well‐being’ ‘Religiosity and spirituality were statistically significant as negative predictors of depression among all ethnic groups’
16	The experience of landscape: Mental health benefits of lakeshore settings	Bentley [42]	USA	2012	Analytical	III‐3	Nature: admiration, connection to place	‘There were statistically significant improvements in mental health scores related to feelings of Alertness and Happiness at all sites, even after controlling for pre‐visit psychological state. Both place attachment and certain restorative setting qualities (notably Fascination/Coherence) made significant contributions toward explaining the variance in mental health scores’
17	Assumptions associated with mental health literacy training—Insights from initiatives in rural Australia	Anderson and Pierce [43]	AUS	2012	Interventional	IV	Community: football clubs Personnel: sports coaches	‘Participants reported increased knowledge of key mental health conditions, increased confidence to help others experiencing mental ill health and less stigmatising attitudes toward mental ill health’ ‘Some leaders, such as teachers, sports club coaches, community leaders and workplace leaders may have more opportunity than others within their professional role to apply newly acquired knowledge and demonstrate increased confidence’
18	Intentional teams in rural health care: A preliminary report	Gudkovs [44]	AUS	2011	Interventional	IV	Community: task sharing Personnel: yoga teacher, personal trainer, psychologist, dietician and occupational therapist	‘Significant improvements in mental health as measured by DASS and SF36 were achieved’ ‘Utilising community resources effectively can lessen the burden on other aspects of the health care system’
19	Resilience in rural community‐dwelling older adults	Wells [45]	USA	2009	Analytical	IV	Intrapersonal: resilience	‘This study found that perceived…mental health status [was] correlated with resilience, and this is well supported in the literature. Mental health status had the strongest association with resilience, and multiple studies support this relationship’
20	Impact of family and social interaction on depressive symptoms among older adults in a rural environment	Bayer [46]	USA	2007	Analytical	IV	Nature: fishing Interpersonal: family, friends Community: church, community support	‘I go to church, it helps me feel better’ ‘Sometimes when I feel bad I call my friend in Dubach. She listens and we talk for a long time. It helps to let it out. Of course it helps to come up here and talk to you and the other patients. It helps to know that I am not the only one having these problems’
21	The influence of stigma and attitudes on seeking help from a GP for mental health problems: A rural context	Komiti et al. [47]	AUS	2006	Analytical	IV	Intrapersonal: positive attitude toward help seeking Personnel: general practitioners	‘Seeking help from a GP for psychological problems was predicted by having a positive attitude toward seeking professional psychological help as well as believing a GP would be helpful in treating psychological problems’ ‘Seventy‐eight individuals had sought [psychological support] from a GP’
22	Impacts of community resilience on the implementation of a mental health promotion program in rural Australia	de Deuge et al. [48]	AUS	2020	Analytical	IV	Intrapersonal: resilience, flexibility and sense of belonging Community: community resilience, connection, resources/infrastructure, flexible delivery and local community event	‘The survey results indicated the primary community resilience strengths across the four sites were related to the connection and caring domain, with most survey respondents agreeing that people in their communities feel like they help each other and that they have a sense of belonging. These survey results were supported by qualitative data, particularly the community mobilisation sub‐theme that emphasised the positive benefits of communities facilitating mental health promotion events and generating conversations about suicide’
23	Belonging and inclusivity make a resilient future for all: A cross‐sectional analysis of post‐flood social capital in a diverse Australian rural community	Matthews et al. [49]	AUS	2020	Analytical	IV	Intrapersonal: personal social cohesion, optimism Community: social connectedness, social networks	‘Informal social connectedness and belonging were important factors for all participant groups, associated with reduced risk of psychological distress’ ‘Belonging and optimism were significantly associated with less ongoing distress for respondents in financial hardship’
24	Stressors, coping resources and depressive symptoms among rural American Indian older adults	Roh et al. [50]	USA	2015	Analytical	IV	Interpersonal: social support Intrapersonal: spirituality Community: culture, community	‘Depressive symptoms were predicted by higher scores on perceived social support and lower scores on functional disability’ ‘Depressive symptoms correlated in a positive direction with functional disability, and in a negative direction with social support and spirituality’
25	Participation in rural community groups and links with psychological well‐being and resilience: a cross‐sectional community‐based study	Lyons et al. [51]	AUS	2016	Analytical	IV	Intrapersonal: self‐efficacy Community: group participation	‘…significant links between particular group characteristics and individual psychological well‐being and resilience, suggesting that the characteristics of the group that an individual participates in are strongly tied to that person's well‐being outcomes’ ‘Multivariable analyses revealed two significant independent factors. First, psychological well‐being was greatest among those who participated in groups without a hierarchy, that is, equal‐status relationships between members. Second, resilience was greater among those who reported having a sense of influence within a group’
26	Finding paradise within: How spirituality protects palliative care clients and caregivers from depression	Penman [52]	AUS	2018	Analytical	IV	Nature: admiration Intrapersonal: spirituality, hope, optimism and acceptance Interpersonal: family support	‘My spirituality makes me feel powerful [despite having cancer] as it gives me strength and courage to persevere in life’ ‘It appeared that spirituality propelled individuals into positive actions that would help them in their present circumstance of confronting death and dying. Both clients and caregivers, viewed spirituality as a human value, whereas engaging in spiritual matters or spiritual engagement referred to some action, which extended also in protecting themselves against depression’
27	Exploring social support, sport participation and rural women's health using photovoice: The Manitoba experience	Scruby et al. [53]	CAN	2019	Analytical	IV	Community: curling club, social connections	‘The women's dialogue and photographs highlighted the importance of curling to mental health’ ‘The women acknowledged the importance of emotional health to their well‐being and valued the opportunity to be present and engaged with other women at the curling club, which provided a respite from stress’
28	Rural men and mental health: Their experiences and how they managed: Feature Article	Gorman et al. [54]	AUS	2007	Analytical	IV	Intrapersonal: inner strength, access to information, sense of humour, taking ownership/self‐efficacy and positive thinking Interpersonal: family support, friend support Community: club involvement, community activities	‘Positive thinking was identified by a number of men as important particularly in relation to the positive aspects in life rather than the negatives. This did not mean denying the negative factors; rather, they recognised the potential for negative feelings to dominate and therefore exclude positive feelings’ ‘Positive thinking was also an activity of looking for and valuing the individual's characteristics’

^a^ NHMRC evidence hierarchy [26].

The four interventional studies varied in methodology and were an evaluation of the effectiveness of mental health first aid training for rural extension agents [3], a case study of a quilt making project within the church [7], findings from a mental health literacy program for a sports team [17], and a 12‐week collaboration program for allied health practitioners [18] (see Table 1 for numerical study identification). These four studies almost exclusively examined strengths in the community and personnel domains of the Socioecological Framework (apart from one study that identified some intrapersonal level strengths). Community level strengths were consistently the most identified strength type throughout the 24 analytical papers.

### 3.3 Evidence for Causal Relationships

The strength of evidence of each study was assessed against the NHMRC evidence hierarchy [26]. In Table 1, 19 studies were classified as level IV (evidence obtained from case series, either post‐test or pre‐test/post‐test) and nine studies were classified as level III‐3 (evidence obtained from comparative studies with historical control, two or more single arm studies, or interrupted time series without a parallel control group).

Across all analytical and interventional studies, only two analytical studies proposed a causal relationship (studies 24 and 25, see Table 1). However, neither had a level of evidence that established a casual relationship [26]. Despite interventional studies having a design that could potentially propose and test a causal relationship, none of the four identified interventional studies obtained strong enough evidence and did not have a comparison group to control for factors other than the strength being causally related to the outcome.

Study 24: Roh et al. [50] examined stressors, coping resources, and depressive symptoms among rural American Indian older adults using an analytical study design. Hierarchical multiple regression was used to test predictors of depressive symptoms and measured perceived social support (family, friends, significant others). The study identified conceptual links to improved mental health from social support, spirituality, culture, and community. A causal relationship was proposed specifically with social support, as the authors found that higher perceived social support was negatively associated with depressive symptoms. Although this relationship could be causal, the analytical study design was classified as a level III‐3 on the evidence hierarchy [26] and did not allow this to be established.

Study 25: Lyons et al. [51] conducted an analytical cross‐sectional household survey of participants in a rural Australian town that examined relationships between group participation and wellbeing. Self‐reported wellbeing and resilience were measured using relevant scales. The study proposed conceptual links to improved mental health from group participation (arts, sports, volunteer groups, etc.) and self‐efficacy. A causal link was proposed specifically for community participation (where participants felt there was no social hierarchy) as this was associated with greater psychological wellbeing and resilience. Again, the study could not establish this relationship as causal as it was limited by the analytical study design and had a level of evidence classification of III‐3 [26].

## 4 Discussion

Rural community strengths are social determinants of mental health [35], however, they are not often considered in the social determinants literature [10]. The results presented in this review suggest there are many untapped assets and resources that research needs to better examine and understand to bridge the gap in mental health. Strengths with the potential to improve mental health were identified across the extent of the Rural Strengths Socioecological Framework continuum, including but not limited to connection to country, spiritual beliefs, family and friend support, social connectedness, community members and groups and clinically trained personnel.

Despite the potential to leverage these strengths across the Rural Strengths Socioecological Framework, the level of evidence to support their use for improving mental health is low in the included studies [26]. The majority of these demonstrated small changes in mental health, and very few studies undertook any kind of intervention that tested the strength of the relationship between a rural strength and mental health. Additionally, the evidence for a causal relationship between the identified strength and improved mental health is limited by the respective study designs. It therefore remains largely unknown how effective it is to integrate strengths into care to improve mental health in rural populations.

High‐quality interventional studies that investigate existing strengths in the natural environment, intrapersonal, and interpersonal levels of the Socioecological Framework are particularly lacking. Nature and intrapersonal strengths were not identified in any interventional studies included in the systematic review, with interventional studies only investigating personnel‐related strengths. This alludes to the supposition that staffing and resourcing are prioritised in higher quality research, and similarly, that a high level of evidence research is less likely to prioritise strengths, such as nature, personal characteristics and social connections.

However, despite the evidence for these strengths‐mental health relationship studies lacking rigour, examples of leveraging were identified throughout the studies. Some of these findings included utilising existing places and people to be mental health advocates [28], social groups acting as protective factors against mental illness [30], and personal beliefs acting as coping strategies [40]. This is promising in that existing assets and resources are being reported as helping the management or prevention of mental health problems. If these strengths could be effectively leveraged, they could contribute to addressing the health care gap.

One implication of these findings is the potential for self‐management strategies to be harnessed. Research suggests that rural populations are familiar with being resourceful and autonomous in the management of their health [55]. As such, this makes them well placed to practise self‐help techniques, especially when access to services is limited or for those who have a reluctance to seek formal help. Some examples of self‐management that came out of the findings of this study include positive thinking and valuing oneself, spirituality, participation in groups, and connecting with country. With that, initiatives to improve mental health literacy in rural locations are much needed [56] and could help to educate and support the use of positive self‐management strategies for mental health. Improving mental health literacy would also encourage positive help‐seeking behaviours [57].

A dominant theme found across studies was the importance of feeling socially connected and supported. Social connectedness is a well‐established protective factor against mental ill‐health [58, 59] and the findings in this secondary analysis presented an array of ways that this strength appeared and was used in the local environment. Rural communities are often described as having good social connections and cohesion [59], however, for those who do not feel they fit in or have a strong sense of belonging, they can experience further isolation [60]. Those who do not feel socially connected may benefit from targeted initiatives to improve social involvement in their local community [51, 60]. Social prescribing, as discussed earlier in this study, has been gaining popularity [10, 12] and may be feasible, acceptable and relevant for rural populations [11]. That said, social prescription faces barriers to uptake, so mapping existing groups and clubs, psychosocial support services, volunteering opportunities, local events and initiatives within the region would aid this. In practice, clinicians and other trusted community members could assist in identifying and referring social prescription to those in need toward opportunities for socialising and community engagement, as task sharing and interdisciplinary collaboration were also found to be important themes in managing mental health problems in rural locations. Coordinating care and collaborating between care disciplines has been encouraged [6, 14] and this would also benefit from service mapping.

Overall, the identified rural strengths in this secondary review show promise for a more holistic approach to mental health support for rural populations and suggest the importance of policy to acknowledge these alternate, nonclinical solutions as meaningful. Taking a strengths‐based approach to identifying assets, resources and protective factors will help to address healthcare gaps by mapping existing supports (which could be aided by the use or application of the Rural Strengths Socioecological Framework). Involving community members, healthcare providers, and stakeholders in this process is critical to identifying relevant strengths, leveraging and coordinating local service providers, and addressing place‐specific needs [6, 14, 61]. To facilitate this, however, a stronger evidence base is required to be implemented and to establish the effectiveness of these strategies before they should be implemented at scale.

### 4.1 Implications for Research and Public Health

Despite the many strengths identified and numerous conceptual links proposed, there are few studies that demonstrated causal pathways or have intervened using rural strengths to improve mental health. Research is needed to better establish the evidence base for strength‐based interventions to be included in mental health policy and planning. Future research should explore strengths across more of the Rural Strengths Socioecological Framework domains, focusing on establishing a causal relationship between strengths and improved mental health and how to harness them effectively. A stronger evidence base is essential for adopting and integrating rural strengths into mental health policy, planning, and investments. The context and acceptability of strengths must be understood to provide better mental health care solutions that are relevant, sustainable and deemed important by rural populations.

### 4.2 Limitations

This study is limited by the strengths and contexts of each paper and thus may not be relevant in other settings. Generally, interventional studies introduce and test an intervention on a group not previously exposed to that intervention. In a rural environment, intervention studies often report on interventions introduced into a rural community from an urban environment. These studies were excluded in the primary analysis as they did not meet the inclusion criteria that required the strengths to be an existing asset/resource in a rural community. Furthermore, longitudinal studies and randomised controlled intervention trials are expensive and few have been published in this area. The literature was gathered from the US, Canada and Australia and may not be generalisable to the rest of the world or, within those countries, may not be transferable to other regions. It is also likely that more causal and interventional research has been conducted in other countries that this review was unable to identify.

## 5 Conclusions

A strengths‐based approach to rural mental health research may provide more relevant solutions to the rural health divide. This study investigated the evidence linking strengths to improved mental health, and examined if and how such strengths have been successfully harnessed. The literature identified conceptual links between many different strengths and improved mental health across the Rural Strengths Socioecological Framework. Although access to social supports and participation in community groups were identified as strengths that should be harnessed, few studies proposed causal relationships. Due to limitations in research design, no study in this review provided a strong enough level of evidence for a causal relationship between a strength and improved mental health. A more robust evidence base is required to support and validate the incorporation of rural mental health strengths into health planning and funding models. Rigorous, high‐quality research that establishes the use of existing community strengths to enhance rural mental health has untapped potential to improve the mental health of rural populations.

## Author Contributions

Annika Luebbe: study conceptualisation and design, analysis of data, review and quality assessment of articles, original draft preparation and editing. Sandra Diminic, Zoe Rutherford, and Harvey Whiteford: assistance in study conceptualisation and design, editing and critical review. Hannah Roovers and Mikesh Patel: assistance in analysis of data, assistance in editing and critical review.

## Ethics Statement

The authors have nothing to report. Ethical approval was not required.

## Conflicts of Interest

The authors declare no conflicts of interest.

## Data Availability

The data that support the findings of this study are available from the corresponding author upon reasonable request.

## (Primary Analysis) Systematic Literature Review Search String

(Rural*[tiab] OR Regional*[tiab] OR Remote*[tiab] OR ‘Rural population’[tiab] OR farm*[tiab] OR ‘Rural Health Services’[MeSH Terms] OR ‘Rural Population’[MeSH Terms] OR ‘rural community’[tiab] OR farms[MeSH Terms] OR ‘Rural Health’[tiab]) AND.

(Mental*[tiab] OR resilien*[tiab] OR ‘community support’[MeSH Terms] OR ‘community participation’[MeSH Terms] OR ‘adaptation, psychological’[MeSH Terms] OR ‘health promotion’[MeSH Terms] OR ‘attitude to health/ethnology’[MeSH Terms]) AND.

(Strength*[tiab] OR facilitate*[tiab] OR ‘protective factor*’[tiab] OR resilien*[tiab] OR opportuni*[tiab] OR ‘protective factors’[MeSH Terms] OR ‘problem solving’[tiab] OR ‘protective factors’[tiab]) AND.

(Australia[mh] OR Australia* OR ‘Northern Territory’ OR Tasmania OR ‘New South Wales’ OR Victoria OR Queensland OR ‘South Australia’ OR ‘Australian Capital Territory’ OR ‘Western Australia’ OR Canada[mh] OR Canada* OR Newfoundland OR Labrador OR ‘Prince Edward Island’ OR ‘Nova Scotia’ OR ‘New Brunswick’ OR Quebec OR Ontario OR Manitoba OR Saskatchewan OR Alberta OR ‘British Columbia’ OR ‘Northwest Territories’ OR Yukon OR Nunavut OR ‘United States’[mh] OR ‘United States’ OR USA OR America[mh] OR America* OR Alabama OR Alaska OR Arizona OR Arkansas OR California OR Colorado OR Connecticut OR Delaware OR Florida OR Georgia OR Hawaii OR Idaho OR Illinois OR Indiana OR Iowa OR Kansas OR Kentucky OR Louisiana OR Maine OR Maryland OR Massachusetts OR Michigan OR Minnesota OR Mississippi OR Missouri OR Montana OR Nebraska OR Nevada OR ‘New Hampshire’ OR ‘New Jersey’ OR ‘New Mexico’ OR ‘New York’ OR ‘North Carolina’ OR ‘North Dakota’ OR Ohio OR Oklahoma OR Oregon OR Pennsylvania OR ‘Rhode Island’ OR ‘South Carolina’ OR ‘South Dakota’ OR Tennessee OR Texas OR Utah OR Vermont OR Virginia OR Washington OR West Virginia OR Wisconsin OR Wyoming).
